# The transcriptome of the olm provides insights into its evolution and gene expression

**DOI:** 10.1038/s41598-025-10073-3

**Published:** 2025-08-03

**Authors:** Susanne Holtze, Defne Demirtürk, Oliver Ohlenschläger, Simon Hegele, Kanstantsin Siniuk, Silke Förste, Venket Raghavan, Marco Groth, Martin Bens, Nils Hassel, An Martel, Maja Lukač, Ivan Cizelj, Martin Fischer, Hequn Liu, Thomas B. Hildebrandt, Steve Hoffmann, Arne Sahm

**Affiliations:** 1 Department of Reproduction Management, Leibniz Institute for Zoo and Wildlife Research, Berlin, Germany; 2 Sahm Lab, Faculty of Biology and Biotechnology, Ruhr University Bochum, Bochum, Germany; 3 CS Protein Production, Leibniz Institute On Aging-Fritz Lipmann Institute (FLI), Jena, Germany; 4 Hoffmann Lab, Leibniz Institute On Aging-Fritz Lipmann Institute (FLI), Jena, Germany; 5 Institute for Medical Informatics, Statistics and Epidemiology, University of Leipzig, Leipzig, Germany; 6 Sahm Lab, IUF-Leibniz Research Institute for Environmental Medicine, Düsseldorf, Germany; 7 Core Facility Next Generation Sequencing, Leibniz Institute On Aging-Fritz Lipmann Institute (FLI), Jena, Germany; 8 Faculty of Veterinary Medicine, Wildlife Health Ghent, Ghent University, Salisburylaan 133, 9820 Merelbeke, Belgium; 9 Faculty of Veterinary Medicine, University of Zagreb, Heinzelova 55, 10000 Zagreb, Croatia; 10 Zagreb Zoo, Fakultetsko Dobro 1, 10000 Zagreb, Croatia; 11 Freie Universität Berlin, Veterinary Medicine, Berlin, Germany

**Keywords:** Molecular evolution, Computational biology and bioinformatics, Evolution, Gene expression

## Abstract

The olm (*Proteus anguinus*), with a predicted maximum lifespan of more than 100 years, is the longest-lived amphibian, which in addition possesses a range of unique adaptations to its dark, subterranean cave habitat. To assess the underlying molecular signatures, we present the first comprehensive transcriptome of the olm. Our study provides gene expression data across six organs and comparative genomics analyses, accessible via an interactive web server: http://comp-pheno.de/olm. The data uncover significant organ-specific gene expression, with the brain showing the highest number of organ-specific expressed genes. Our findings reveal significantly more genes under strong negative selection than positive selection, particularly in brain-specific expressed genes. Processes under positive selection in the olm resemble those in other long-lived species.

## Introduction

The olm (*Proteus anguinus*) is Europe’s only cave-dwelling vertebrate and the only representative of the *Proteus* genus. Olms – as several further amphibian species, especially in the order Caudata—display neoteny, i.e. they retain juvenile characteristics into their adult life. In olms, these characteristics comprise reduced ossification, and the lifelong retention of a tail and of gills, enabling an exclusively aquatic lifestyle^1^. Unlike in the neotenous Axolotl (*Ambystoma mexicanum*), in olms the artificial induction of metamorphosis has not been accomplished^2^. In general, neoteny has been associated with longevity, which is consistent with an average lifespan of 68 years and a predicted maximum lifespan of more than 100 years^3^. Despite their small body size – they reach a length of 30 cm weighing up to 35 g—this even renders them the longest-lived of all amphibian species and a highly interesting alternative animal model of ageing^4^. Olms exhibit a relatively low metabolic rate^5^, however, the causes and molecular mechanisms underlying their longevity are largely unknown. Genomic and transcriptomic studies of amphibians and especially salamanders are challenging due to their large genome size and comparably limited annotation resources; until recently, the 32-gigabase-pair axolotl genome was the largest genome to be sequenced^6^. The genome of the olm is estimated to be even in the range of 50 gigabases^7^.

To address these challenges, we generated a hybrid transcriptome assembly combining Illumina short-read accuracy with Oxford Nanopore long-read contiguity. This approach is more likely to resolve full-length isoforms and low-abundance transcripts potentially critical for identifying longevity-associated pathways, while circumventing biases inherent to either technology alone^8,9^.

## Methods

### Sample collection

The three olm individuals used in this study originated from the Vedrine area (Sinj) in middle Dalmatia (Croatia). They had been found flushed from caves during the strong spring currents and were then transferred to Ghent University (Belgium). Individuals were allowed to acclimatize for 6 weeks. They were housed singly at 11 °C in complete darkness in aquaria containing 3 L of aged tap water and a hiding place. Once a week, the water was replaced and animals fed with tubifex. The animals were clinically healthy and tested negatively for *Batrachochytrium salamandrivorans*, *B. dendrobatidis* and Ranavirus as assessed using qPCR on skin swabs. The three olms whose organs were used in this study served as non-infected control animals to further six ones that were experimentally infected with the *B. salamandrivorans* type strain (AMFP13/1) to assess susceptibility to the disease^10^. Animals were euthanized after 6 months with an intraperitoneal pentobarbital injection. Inner organs were extracted immediately upon death and stored in RNAlater (Thermo Fisher Scientific) at −80 °C. A sample set of six organs, brain, gut, heart, liver, lung, and skin was collected from the three individual olms.

Since 2012, the Zoo of Zagreb has been officially permitted to collect and house olms, which are flushed out in a natural way in the area specified above. These flushed out animals represent a natural minus for the population, as there is no possibility for them to return to the cave system due to the morphology of the terrain. Animal experimental procedures were approved by the ethical committee of the Faculty of Veterinary Medicine (Ghent University) (EC2017/75). This study is performed in accordance with relevant guidelines and regulations. All methods are reported in accordance with ARRIVE guidelines.

### Sequencing

After homogenizing samples using TissueLyser (Qiagen), standard TRIzol RNA extraction was applied followed by DNase I digest and purification Direct-zol RNA Micro Prep Kit (Zymo). RNA was extracted using the Norgen RNA Purification Kit. RNA-seq libraries were prepared using the NEBNext Ultra II Kit. Libraries were prepared from 500 ng of input material (total RNA) using NEBNext Ultra II Directional RNA Library Preparation Kit in combination with NEBNext Poly(A) mRNA Magnetic Isolation Module and NEBNext Multiplex Oligos for Illumina (Unique Dual Index UMI Adaptors RNA) following the manufacturer’s instructions (New England Biolabs). Quantification and quality assessment of libraries were performed using a TapeStation 4200 instrument and a D5000 ScreenTape (Agilent Technologies). Libraries were pooled and sequenced on one NovaSeq 6000 SP 200 cycle run. Sequencing resulted in around > 60 million read-pairs per sample.

Additionally, RNA samples from all tissues of a single individual (p4) were pooled and a barcoded library was prepared using PCR-cDNA Barcoding Kit (SQK-PCB109, Oxford Nanopore Technologies, Oxford, UK) from 50 ng total RNA according to manufacturer’s protocol, with 20 min PCR extension time and 16 cycles. The library was sequenced on R9.4.1 flow cell (FLO-MIN106D; 1,745 pores) using ONT MinION Mk1B device and MinKNOW Software (v23.04.5) for 72 h. Data extraction was performed using Guppy (v6.5.7, Fast model, 450 bps), yielding 9.66 million high-quality RNA-seq long-reads (6.18 Gbp) with a minimum read length of 200 bp.

### Read preprocessing

Illumina short-reads were adapter-clipped and quality-trimmed using Cutadapt v4.9^11^ and Trimmomatic v0.39^12^. Oxford Nanopore Technologies’ long-reads were error corrected by feeding them together with the preprocessed Illumina short-reads of the matching individual into Ratatosk v0.9.0^13^.

### Assembly and annotation

A hybrid transcriptome assembly was created using Trinity v2.15.1 with the “–long_reads” parameter, “–max_memory 500G” and, otherwise, default settings^14^, based on the corrected long-reads and the short-reads of the same individual. Open reading frames were extracted using TransDecoder v5.5^15^. Functional annotation was performed using the eggNogMapper 2.1.11^16^ with the following parameters: “-m diamond –evalue 0.001 –score 60 –pident 40 –query_cover 20 –subject_cover 20 –itype CDS –translate –tax_scope auto –target_orthologs all –go_evidence non-electronic –pfam_realign none”**.**

### Transcriptome completeness

The completeness of the olm transcriptome and other public amphibian transcriptomes was assessed using BUSCO v5.4.5^17^ with parameters set for vertebrate single-copy orthologs. The following additional species were analyzed with the respective NCBI Transcriptome shotgun ID and Bioproject ID in parentheses: *Ambystoma mexicanum* (GFZP01, PRJNA378982), *Hynobius chinensis* (GAQK01, PRJNA229834), *Batrachuperus yenyuanensis* (GHDZ01, PRJNA515112), *Taricha granulosa* (GHKF01, PRJNA505885), *Bolitoglossa vallecula* (GHME01, PRJNA419601), *Salamandra Salamandra* (GIKK01, PRJNA607429), *Tylototriton wenxianensis* (GESS01, PRJNA323392).

### Positive selection analysis

The haplotype caller of GATK v4.2.1 with default settings^18^ was used to identify variant sites in the three individuals examined. Subsequently, bcftools v1.19^19^ was used to replace nucleotides at variant sites in the transcriptome sequence with ‘N’ characters. Positive selection analysis was conducted using PosiGene v0.1^20^ with the olm both as reference and anchor species and “-min_ident = 70.00” requiring each orthologous sequence to show at least 70% identity with the respective olm sequence. PosiGene calls internally Gblocks v 0.91b^21^ with “t = c”, “b4 = 30”, and “b2” set to the number of sequences (= species) in the alignment. Besides the coding sequences of the olm, public coding sequences of the amphibian species mentioned above in the ‘Genome completeness’ sub-chapter were used as input. HA.foreground.omega values (*d*_N_/*d*_S_) were used as input for a gene set enrichment analysis using the R clusterprofiler package^22^. The resulting gene ontology terms were further summarized using the rrvgo R package^23^. Genes showing overall *d*_N_/*d*_S_ > 1 for the olm with PosiGene (branch-site test) were further validated using hyphy v2.5.8 aBSREL on the respective alignment. Overall *d*_N_/*d*_S_ ratios were calculated by adding the products of the *d*_N_/*d*_S_ values of each rate class with the proportion of the respective rate class. Only genes showing *d*_N_/*d*_S_ > 1, both, with PosiGene (branch-site test) and hyphy aBSREL were reported as potentially positively selected.

### Gene expression analysis

For each gene that was functionally annotated, the longest transcript was chosen as its representative. Preprocessed reads were mapped against this filtered reference using bwa mem^24^. Quantification was performed using featureCounts v2.0.3^25^. Organ-specific genes were determined by testing the samples of the respective organ against all other samples using DeSeq2 v1.34^26^. Fold-changes were adjusted using the lfcshrink function, with the apeglm shrinkage estimator. A gene was counted as organ-specific if it had an FDR < 0.05 and a higher gene expression with a fold-change > 2. Fold-Changes were used as input for a gene set enrichment analysis using the R clusterProfiler package v4.2.2^22^.

### Protein structure predictions

Structure predictions of NDUFS4 were performed using the AlphaFold3 web server with default options^27^. For graphical representations the program ChimeraX^28^ was used. The Matchmaker algorithm as implemented in ChimeraX was employed to calculate the superimpositions of the predicted models of human and olm NDUFS4 with the recently released cryo-EM model of *Mus musculus* NDUFS4 in the I2 + III2 supercomplex^29^ (PDB code 8UCA).

## Results and discussion

We sequenced 14 olm samples covering six organs across three individuals using Illumina short-read technology. Additionally, we employed Oxford Nanopore technology to obtain long-reads from one individual (Table 1, Supplement Table S1).

**Table 1:** Illumina RNA-seq conducted in the olm (each > 60 million reads per sample).

Tissue	Brain	Gut	Heart	Liver	Lung	Skin
# Samples	2	2	2	3	3	2

### De novo transcriptome assembly of the olm

We performed a hybrid transcriptome assembly by combining Oxford Nanopore Technologies’ long-reads with Illumina short-reads. Initially, the error-prone long-reads were corrected using the more accurate short-reads with Ratatosk^13^. Then both long-reads and short reads were fed into Trinity^15^ resulting in 541,591 assembled transcripts (N50 = 1,402 bases) that were classified by Trinity into 370,474 ‘gene’ groups. We extracted open reading frames using TransDecoder^15^. Functional annotation with eggNogMapper^16^ identified 40,749 annotated transcripts (N50 = 3,313 bases) corresponding to 18,924 protein-coding genes (5.1% of Trinity’s original ‘gene’ groups, see above). All subsequent analyses were conducted with this filtered set of sequences.

**Fig. 1:**
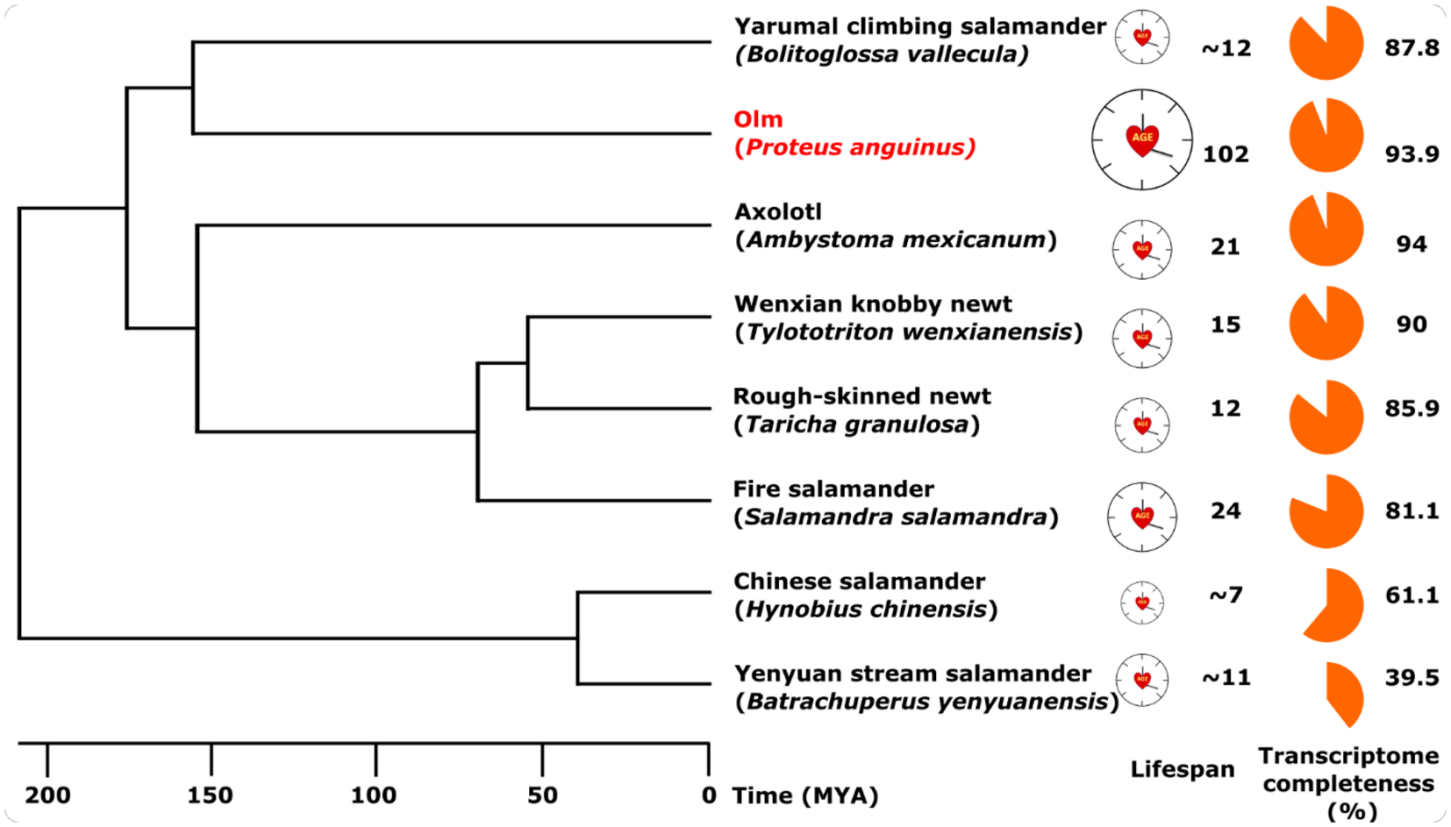
BUSCO-completeness of olm (*Proteus anguinus*) transcriptome assembly in comparison to other Amphibian (Caudata) transcriptomes available at NCBI. Lifespans were taken from AnAge^30^.

The completeness of the transcriptome was estimated at 93.9% using BUSCO, based on conserved single-copy vertebrate genes (see Fig. 1 for a comparison with other publicly available amphibian transcriptomes on NCBI). Although no genome or transcriptome for the olm is available on NCBI, we identified one study containing an olm transcriptome assembly in its supplemental data (Bartas et al. 2021). However, this transcriptome is based on only 5.6 million reads, resulting in a completeness of 27.4% as estimated by the same BUSCO approach used for other assemblies (cf. Fig. 1).

### Gene expression atlas

**Fig. 2:**
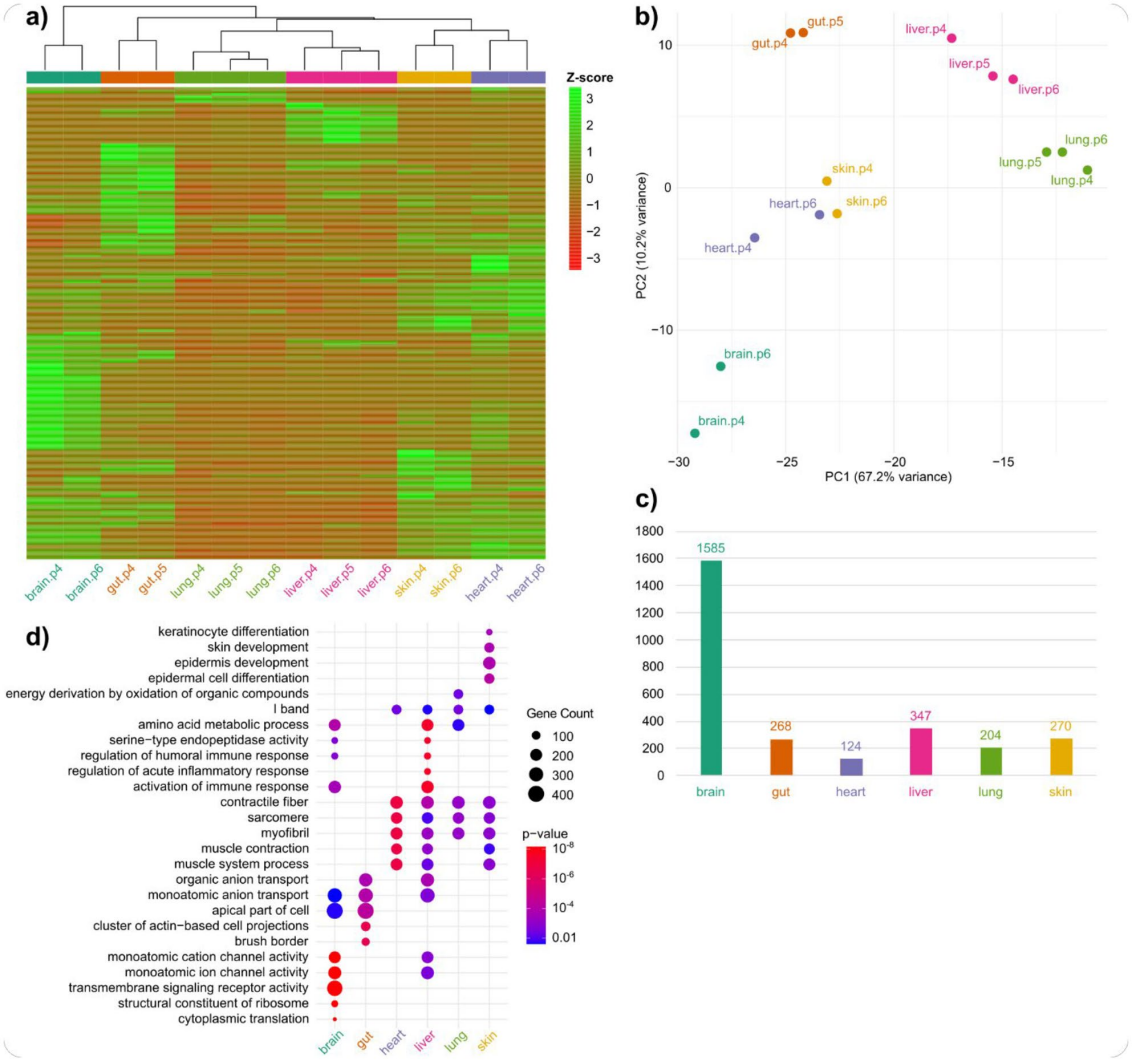
Gene expression in the olm across organs examined. (**a**) Clustering of samples based on the most highly expressed genes (transcript per million value of > 100, n = 723). “p4”, “p5” and “p6” denote different olm individuals examined. (**b**) Principal component analysis using the same data as in a. (**c**) Number of organ-specific genes, i.e. differentially expressed in comparison to all other respective samples (FDR < 0.05, Fold-Change > 2). (**d**) Summary of gene set enrichment analysis of organ-specific genes. Displayed are the five terms per organ with the lowest p-value respectively. Four terms belong to the top-5 of multiple organs resulting in the 26 terms displayed.

Next, we determined the expression levels of all genes for which transcripts could be reconstructed (Supplement Table S2). Clustering the samples by expression levels showed that samples of the same organ were always more similar to each other than to other respective samples (Fig. 2a). A principal component analysis (PCA) yielded similar results, although heart and skin samples exhibited high similarity to each other in this analysis. Brain samples showed the least similarity to samples of other organs in both clustering and PCA (Fig. 2b).

We then identified genes that were significantly higher expressed in each organ compared to the combined samples of all other organs (organ-specific genes, Supplement Tables S3-S8). Consistent with the clustering and PCA results, the brain exhibited by far the highest number of organ-specific genes (Fig. 2c). These findings are typical for gene expression studies in mammals. For example, in RNA-seq studies of samples from different individuals, organs, and even species, samples usually cluster first by organ. Brain samples are often outliers in RNA-seq studies examining a limited number of organs and typically show the highest number of organ-specific genes^31–33^.

We also systematically analyzed whether organ-specific genes were enriched for gene ontology terms. Unsurprisingly, many of the most strongly enriched terms represent biological processes or components directly linked to the respective organ. For example, “epidermis development” is linked to skin, “muscle contraction” to heart, and “brush border” to gut (Fig. 2d, Supplement Tables S9-S14). Thirteen out of twenty-six of the most strongly enriched terms are enriched in multiple organs. For example, “activation of immune response” is among the most strongly enriched gene ontology terms in the liver but is also enriched in the brain, albeit with less extreme significance.

Our study is limited by the small number of biological replicates per organ, which is a consequence of the limited availability of samples of sufficient quality. Despite this limitation, the dispersion plot confirms proper model fit, with estimates decreasing along the mean-normalized count trend (Fig. S1). To minimize the occurrence of false positive organ-specific genes, we additionally applied a filtering criterion with a fold-change requirement of at least 2, in addition to an FDR < 0.05 (see Methods). While simulations indicate that DESeq2 maintains robust false discovery rate control (< 5%) even with low replicate numbers under these conditions, this rigor likely incurred a 20–40% reduction in true positive detection rates, resulting in substantial false negatives^34^. Future research should not only increase the number of biological replicates but also include a broader set of organs to determine whether other organs, besides the brain, have specifically expressed genes that are subject to strong selection pressures.

### Analysis of positive and negative selection

We conducted an analysis of positive and negative selection, comparing the coding sequences of the olm with orthologs in other amphibian transcriptomes (Fig. 1). After applying PosiGene’s orthology pipeline, we retained 3,488 genes for downstream analysis. Using the branch-site test of positive selection^35^, we identified one gene, namely *COL4A5*, as having a significant p-value (< 0.05) after multiple test correction. This gene encodes the α5-chain of type IV collagen, an essential component of extracellular basement membranes in epithelial organs^36^. An intact *COL4A5* gene is crucial for kidney filtration and hearing, as mutations can lead to membrane alterations that impair or destroy these functions^37^. Moreover, when genes are ranked by their selection p-values (Supplement Table S15), the next top-ranked genes, however, without significance after multiple test correction, are *BASP1* (also known as CAP23) and *VIM* (vimentin). Both genes are known to be functionally linked to collagen and to each other. BASP1 transduces information from extracellular matrix to cytoskeleton inside cells^38^ control expression of collagen II^39^ and can activate mitochondrial apoptosis pathway^40^. Vimentin is an essential part of cytoskeleton that controls cell migration on collagen^41^, mitochondrial membrane potential^42^, in turn, is controlled by BASP1^43^. Collagen IV expression is decreased during aging^44,45^, while the expression of its degrading factors is unaffected^46^. Decreased amount of collagen IV was suggested to be a potential reason for reduced healing capacity in aged^46^. In contrast, vimentin protein levels and stability of vimentin filaments in human fibroblasts are increased with age^47^ and single allele mutation in vimentin gene is a known cause of human progeroid syndrome^48^.

Although our primary approach for detecting positive selection was the branch-site test, which identified only a single gene (*COL4A5*) as significant at FDR < 0.05, it is highly unlikely that just one gene in the olm lineage has been subject to positive selection. The low number of significant results is most likely due to a high rate of false negatives, which can be attributed to several factors: the limited number of available comparative transcriptomes, their incomplete gene models, and the considerable evolutionary distance (over 100 million years) to the closest related species with genomic resources. To address these limitations and provide a broader, albeit less stringent, perspective, we also considered the *d*_N_/*d*_S_ ratio on the olm branch as a supplementary indicator of selection. A *d*_N_/*d*_S_ ratio (non-synonymous to synonymous substitution rates) > 1 is typically interpreted as a indicator of positive selection, while a *d*_N_/*d*_S_ value < < 1 indicates negative (purifying) selection. We found 53 genes with *d*_N_/*d*_S_ > 1 using both PosiGene and aBSREL, suggesting these genes are putatively under positive selection. We found 1,180 genes with *d*_N_/*d*_S_ < 0.1 using PosiGene, indicating putative strong negative selection (Supplement Table S15).

**Fig. 3:**
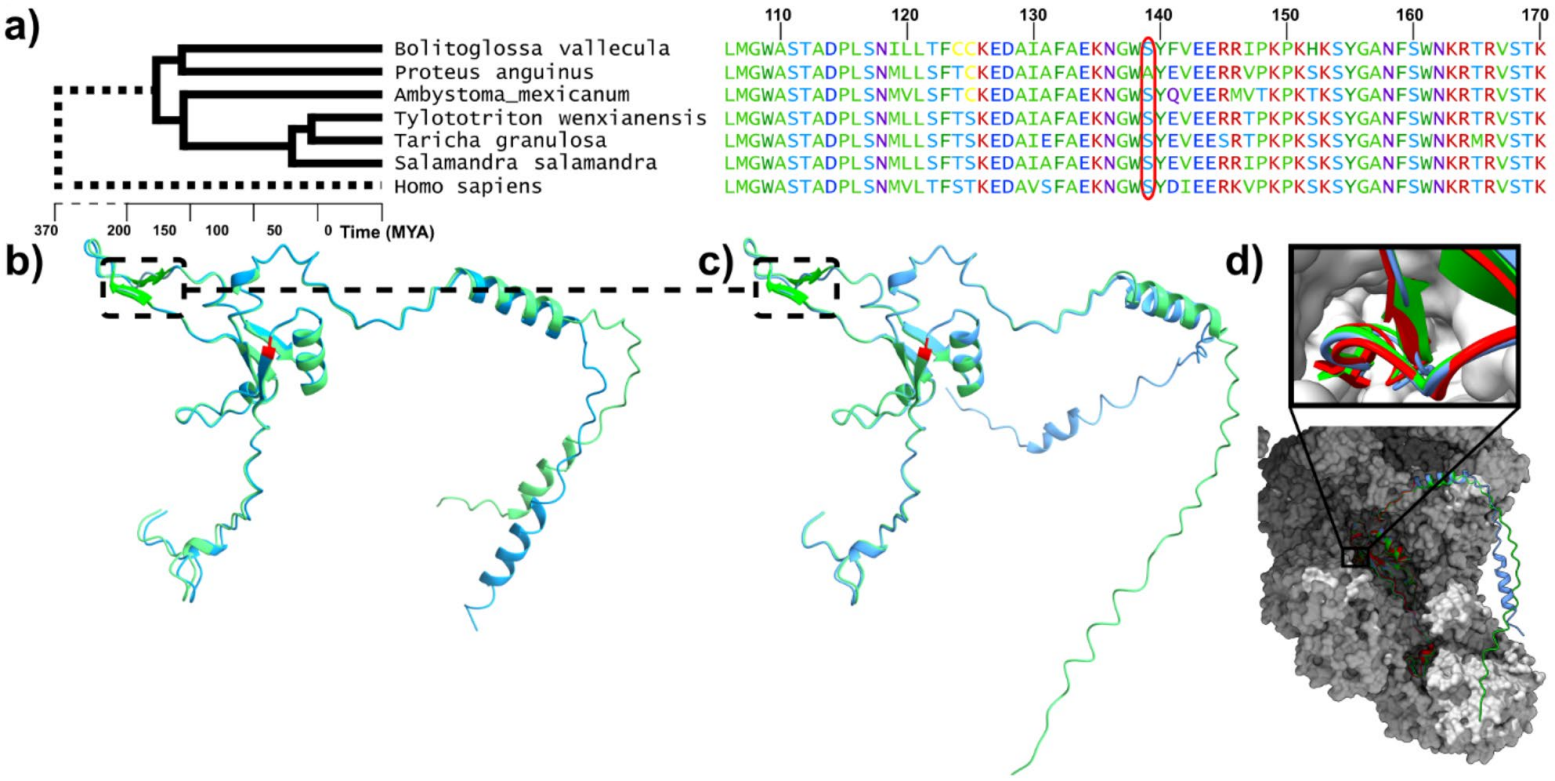
**a**) Displayed is the C-terminal part of the NDUFS4 amino acid sequence alignment for the olm, other amphibian species, and humans. The numbering at the top refers to the sequence position in the olm. The site in the olm that deviates from the consensus sequence, with a positive selection probability of 98.39%, is highlighted (A139). Divergence times for humans and amphibians were taken from^49^. (**b**) Shown in blue is the predicted tertiary structure of the olm NDUFS4 amino acid sequence, with the site predicted to be under positive selection in the olm highlighted in red (alignment position 139, cf. with a). In green, the predicted 3D structure of a modified olm sequence is shown, where the single amino acid identified as positively selected in the olm is replaced by the amino acid present in the other species (S instead of A at alignment position 139). This replacement, according to the prediction, results in the formation of an antiparallel ß-sheet further downstream towards the C-terminus (dashed box). (**c**) In green, the predicted tertiary structure of the human NDUFS4 amino acid sequence is shown. Again, the formation of an antiparallel ß-sheet further downstream in the protein is predicted, similar to the modified olm sequence that matches the human and other species at alignment position 139 (dashed box). The amino acid site at alignment position 139 (cf. with a) in the olm is highlighted in red. If this site is modified to match the original olm sequence at alignment position 139 (A instead of S), as shown in blue, the antiparallel ß-sheet is lost according to the prediction. (**d**) A molecular model of the mouse (*Mus musculus*) I2 + III2 supercomplex derived from cryo-EM 8UCA, not available for human) is shown in grey. The predicted 3D structures of the mouse, human, and olm NDUFS4 subunit are shown in red, green, and blue, respectively. The inset displays details of the deep and narrow cleft hosting a loop between a.a. 99–113 in which the formation of an antiparallel ß-sheet is predicted for mouse and human but not in the olm NDUFS4 protein.

Our web server was implemented to enable detailed examination of individual genes, complementing the broader scope of this article. The server allows users to visualize alignments and positively selected sites, and to rank genes by various statistics. For example, we ranked genes by their *d*_N_/*d*_S_ value and found *NDUFS4* among the top 20, with a *d*_N_/*d*_S_ ratio of 14.6, indicating strong positive selection. This gene stood out because its highly conserved C-terminus had a site with a positive selection probability of 98.39%. At this site, the olm has an alanine (A139) instead of the serine found in all other examined amphibians (Fig. 3a). Alignment of the olm’s NDUFS4 sequence with other species revealed that the serine at this position is conserved even in mammals, including humans (S139).

Next, we assessed the potential structural impact of the mutation in the olm using AlphaFold3^27^. We compared the model of the olm sequence (Fig. 3b, blue) with a modified olm sequence where alanine was replaced by serine (A139S), as found in other species (Fig. 3b, green). According to the structure prediction, this replacement results in the formation of two antiparallel β-strands further downstream towards the C-terminus, which are absent in the original olm sequence (Fig. 3b, dashed box). In structure predictions for NDUFS4 from other amphibians and humans (Fig. 3, green, dashed box), these antiparallel β-sheets are also present. When the human sequence is modified to match the olm at the relevant site (serine replaced by alanine), the antiparallel β-strands disappear (blue, Fig. 3c), similar to the prediction for the original olm sequence.

NDUFS4 is a subunit of the mitochondrial respiratory chain complex I. The structure of the I2 + III2 supercomplex, i.e. complex of two units of Complex I and two units of Complex III, has been elucidated in mouse using cryo-electron microscopy^29^ (PDB code 8UCA). When modeling the secondary structure of the mouse, human, and olm NDUFS4 subunits in the context of the overall structure of the mouse I2 + III2 supercomplex, a similar pattern emerges (Fig. 3d, Fig. S2). Interestingly, the region where the protein structure changes from antiparallel β-strands to unordered in the olm is part of a protrusion of the NDUFS4 protein that fits into a deep and narrow cavity of the rest of the complex (see inset of Fig. 3d). It is possible that the loss of the antiparallel β-strands in the olm leads to increased flexibility and reduced spatial strain of the NDUFS4 protein structure at this position. These predictions suggest that the mutation causes NDUFS4 in the olm to lose two antiparallel β-strands that are otherwise highly conserved during evolution, potentially affecting the interaction of NDUFS4 with the rest of complex I.

NDUFS4 is phosphorylated by AMP-activated protein kinase (AMPK) at serine-173 and such phosphorylation is essential for NDUFS4 import to mitochondria and proper function of complex I^50,51^. Structure prediction based on the recently released cryo-EM model of NDUFS4 in the I2 + III2 supercomplex^29^ shows that the C-terminal phosphorylation site of NDUFS4 (S168^olm^/S173^human^) binds via an extended (and in free state probably intrinsically disordered) stretch E143-Y155^olm^/E148-Y160^human^ into a cleft of the NADH-ubiquinone oxidoreductase 75 kDa subunit (Fig. S3). This strained conformation extending from the ß-strand hosting A139^olm^/S144^human^ allows to anticipate that a mutational change at this conserved position can directly disturb binding at the more distant phosphorylation site. Frameshift and truncating mutations in NDUFS4 cause acute complex I deficiency, lactic acidosis and encephalopathy leading to death of newborns and infants^52,53^. Pharmacological AMPK activation by metformin leads to life extension in nematodes, fruit flies and rodents^54^. Several lines of evidence suggest that the benefits of AMPK activation are achieved through modulation of complex I activity which is known to be dysregulated in several age-related pathologies and aged animal models^55,56^. Of interest, protein levels of NDUFS4 show positive correlation with lifespan of multiple vertebrate species^57^.

**Fig. 4:**
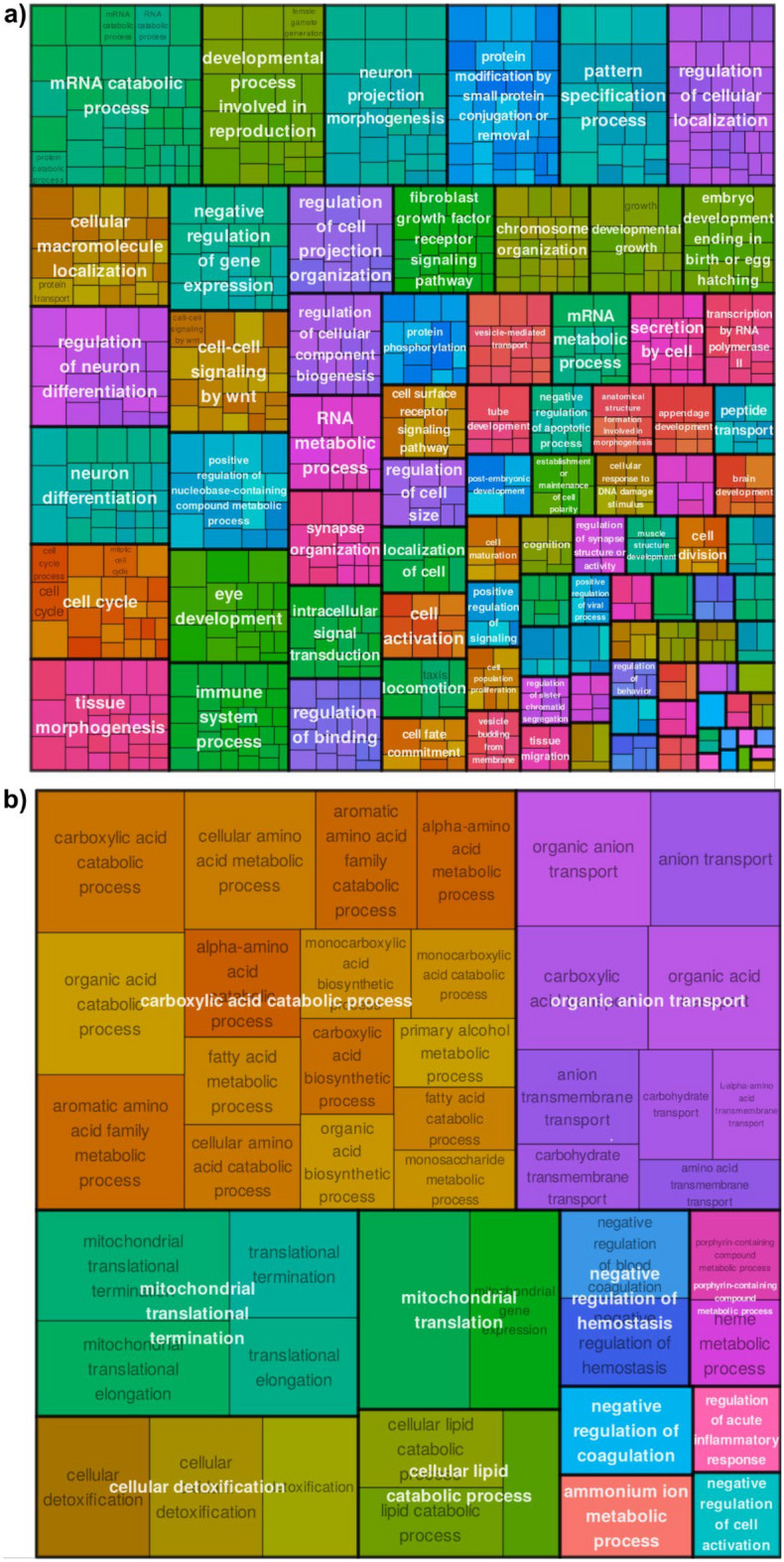
(**a**) Biological processes found to be significantly (FDR < 0.05) affected negative selection (*d*_N_/*d*_S_ < 0.1). (**b**) Biological processes found to be significantly (FDR < 0.05) affected by positive selection (*d*_N_/*d*_S_ > 1).

For a systematic analysis of positive and negative selection on the pathway level, we conducted a threshold-free gene set enrichment analysis (GSEA) using the *d*_N_/*d*_S_ ratios. We found 73 and 1344 GO terms significantly affected by positive and negative selection, respectively, in the olm (Supplement Tables S16,S17). We then summarized the terms using semantic clustering (Fig. 4a,b). Our finding of more genes and processes affected by negative selection than by positive selection aligns with similar results in many cross-species comparisons across various evolutionary clades^58^. This pattern of negative selection is consistent with the critical importance of maintaining the function of these processes across diverse organisms. Processes linked to “development,” “morphogenesis,” and “cell cycle” were under strong negative selection (Fig. 4a), consistent with their known conservation across different model organisms^59–61^. “Neuronal differentiation” processes were also under strong negative selection (Fig. 4a), with many involved transcription factors being known as highly conserved^62^.

Importantly, given the longevity of the olm, several processes linked to “mitochondrial translation” were affected by positive selection (Fig. 4b). Inhibiting mitochondrial translation prolongs the life of mice and worms^63,64^. Mitochondrial translation, as part of mitochondrial biogenesis, was found to be under positive selection in species with extreme lifespans, such as naked mole-rats, killifishes, and clownfishes^65–67^. Adaptation of this process may play a role in the extended lifespan of the olm. Additionally, many “catabolic processes,” such as those involving amino and fatty acids, were under positive selection. These processes, particularly autophagy, are essential for lifespan extension by calorie restriction in different model organisms^68,69^. Adaptation of “cellular detoxification” processes, which eliminate harmful substances like free radicals, may contributes to reduce cellular damage and maintain tissue function over an extended lifespan. Similar enhancements were found in long-lived turtles, bats, clownfishes, and clams. However, our analysis is limited by the lack of a closely related comparison species (Fig. 1). Further research is required to determine whether the processes under positive selection in this study were specifically adapted in the olm or at an earlier evolutionary stage. Furthermore, interpretation of GO enrichment results must be approached with caution, as GO terms can be both broad and imprecise, potentially grouping together genes with distinct or even unrelated functions. Furthermore, direct comparison of the positively selected genes identified in the olm with known mammalian aging-related genes is complicated by the deep evolutionary divergence between these lineages. As a result, while some overlap in pathways is observed, the correspondence at the level of individual genes remains uncertain. Our analysis may miss olm-specific, rapidly evolving genes, as we filtered out 95% of genes that did not receive a functional annotation (see above).

**Fig. 5:**
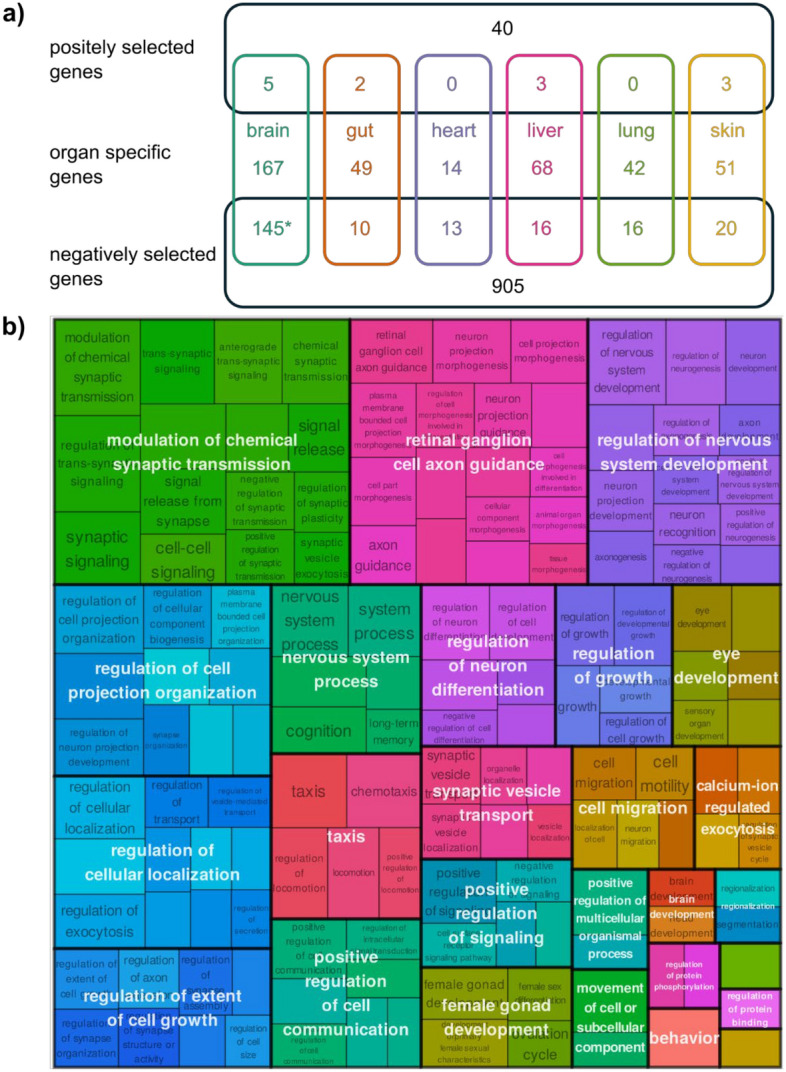
(**a**) Overlap of organ-specific genes with positively and negatively selected genes (*d*_N_/*d*_S_ > 1 and *d*_N_/*d*_S_ < 0.1, respectively). The numbers in the middle represent organ specific genes that were found to be neither positively or negatively selected. Sums deviate from the numbers shown in Fig. 2c as only genes were counted that could be examined in both analyses (gene expression and selection). A “*” represents a significant overlap (Fisher test, p = 0.002 for brain-specific and negatively selected genes). (**b**) Biological processes found to be significantly enriched in the intersection of brain-specific expressed and negatively selected genes.

We investigated the intersections of organ-specific genes with those under positive and negative selection. Notably, we identified a significant overlap between brain-specific genes and negatively selected genes (p = 0.002, Fisher’s exact test, Fig. 5a). Further analysis of this overlap revealed enrichment in several biological processes and components, including “modulation of chemical synaptic transmission,” “regulation of nervous system development,” and “regulation of neuron differentiation” (Fig. 5b). These findings suggested that brain-specific genes, which are crucial for the development and function of the brain, are subject to strong selective constraints, rendering them particularly intolerant to mutations that could disrupt these functions. This phenomenon is not unique to the olm or amphibians; it has been well-documented that neural genes are under intense negative selection in primates^70^. A study comparing 18 human tissues showed that brain-expressed genes display the smallest amount of amino acid changes^71^. Furthermore, genes associated with neuroanatomical phenotypes are under strong negative selection in mice^72^. Our findings suggest that the negative selection of genes crucial for brain function and development may be more widespread in vertebrate evolution. Additionally, several cross-species studies have found a general association between higher gene expression and stronger negative selection^73–75^. In total 13 genes displayed signatures of positive selection and organ-specific gene expression (Fig. 5b, Supplement Table S18). The brain-specific overexpression of DAO (D-amino acid oxidase) and AK1 (adenylate kinase 1) highlights potential adaptations to neural maintenance in the olm. *DAO* induces cellular senescence via reactive oxygen species (ROS) generation^76^. *AK1* optimizes ATP/ADP ratios, ensuring efficient energy homeostasis in metabolically active brain regions^77^. In the liver, *AXIN2*-a key regulator of Wnt/β-catenin signaling-shows elevated expression and selection signatures. Wnt signaling is integral to tissue repair and stem cell maintenance^78^, processes that decline with age in most species.

### Web server

**Fig. 6:**
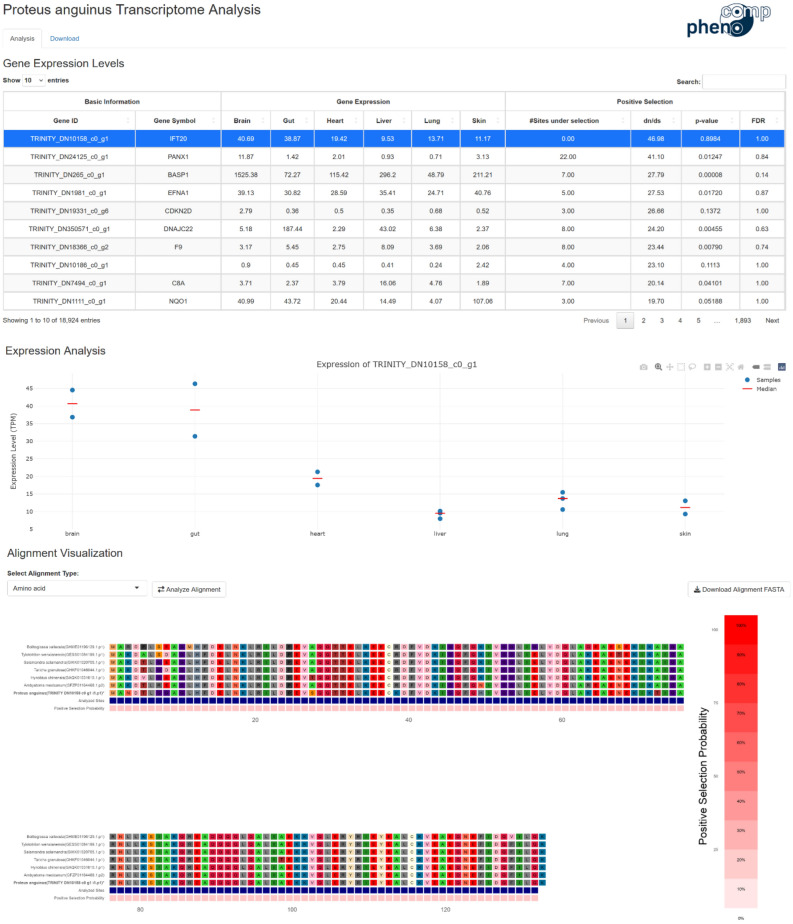
Example view of the olm transcriptome web server. The webserver consists of a table that is searchable and sortable, e.g., by gene expression if different tissues or positive selection analysis results (top). For the gene selected by the user (in this case *IFT20*), the respective expression values (middle) and, if available, the alignment of the olm sequence against the respective orthologs of the other species is shown (bottom).

To make our data easily accessible online, we built an interactive Shiny application. For each gene examined, we provide visualizations of gene expression data across different samples and organs, as well as comparative genomics analyses. The latter includes alignments of the respective olm amino acid or coding sequences against the matching orthologs of other amphibian species, with sites predicted to be positively selected in the olm highlighted (Fig. 6). The data is searchable by gene symbol and sortable by various criteria, such as average expression levels in different tissues or positive/negative selection indicators. The web service is available under http://comp-pheno.de/olm.

## Conclusions

We publish the transcriptome of the olm, providing for the first time genome-wide coding and amino acid sequences. Additionally, we offer gene expression data and make it easily accessible alongside our comparative genomics data through a web server. These resources are crucial for further research into this extraordinary species.

As expected, the gene expression data clusters primarily by organ. The organ-specific expressed genes and signaling pathways closely resemble those of mammals and other well-studied species. Consistent with studies in other species, we find that the brain differs significantly from other organs in its expression profile.

We identified substantially more genes and biological processes under strong negative selection than under positive selection in the olm. Negatively selected genes show a significant overlap with brain-specific expressed genes. This finding aligns with studies in various mammals that have found brain-specific genes to be subject to intense negative selection, suggesting that this phenomenon may be more widespread in vertebrate evolution. Interestingly, the processes under positive selection in the olm largely match those found to be heavily adapted in other long-lived species. This supports the hypothesis that changes in mitochondrial translation, as well as several catabolic and cellular detoxification pathways, could represent convergent evolutionary adaptations favoring longevity.

In the future, it would be valuable to analyze a greater number of biological replicates and a wider range of organs than those available in our current study. Additionally, sequencing data from closely related species, such as the common mudpuppy (*Necturus maculosus*), would be crucial to determine whether the identified mutations are specific to the olm or if they occurred earlier in evolution. This neotenous species of the sister evolutionary branch is the closest relative of the olm, but has a predicted lifespan of only 34 years while occurring at comparable ambient temperatures. Lifespan positively correlates with body mass and negatively with body or in case of ectothermic amphibians with ambient temperature. Such a comparative approach would enable a clearer association of changes in specific genes and signaling pathways with distinct phenotypes, such as the longevity of the olm. Furthermore, cross-species gene expression studies with closely related species could elucidate how changes in gene expression, in conjunction with alterations in amino acid sequences, may have contributed to the evolution of these phenotypes.

## Supplementary Information

Supplementary Information 1.

Supplementary Information 2.

## Data Availability

All raw data were deposited as NCBI BioProject PRJNA1207464. The transcriptome assembly of the olm was deposited as NCBI Transcriptome Shotgun Assembly GLBZ00000000. Processed gene expression data were deposited as Gene Expression Omnibus study GSE286268. The code for the Shiny web server was deposited as GitHub repository asahm/Olm_webserver and the underlying data was deposited as Zenodo repository 15391008.
