# Supplementary material for: The transcriptome of the olm provides insights into its evolution and gene expression
Source: Sci Rep. 2025 Aug 3;15:28324. doi: 10.1038/s41598-025-10073-3 (PMC12319086; doi:10.1038/s41598-025-10073-3)
Supplement: Supplementary file 1 — Supplementary Information 1. [file 41598_2025_10073_MOESM1_ESM.pdf]

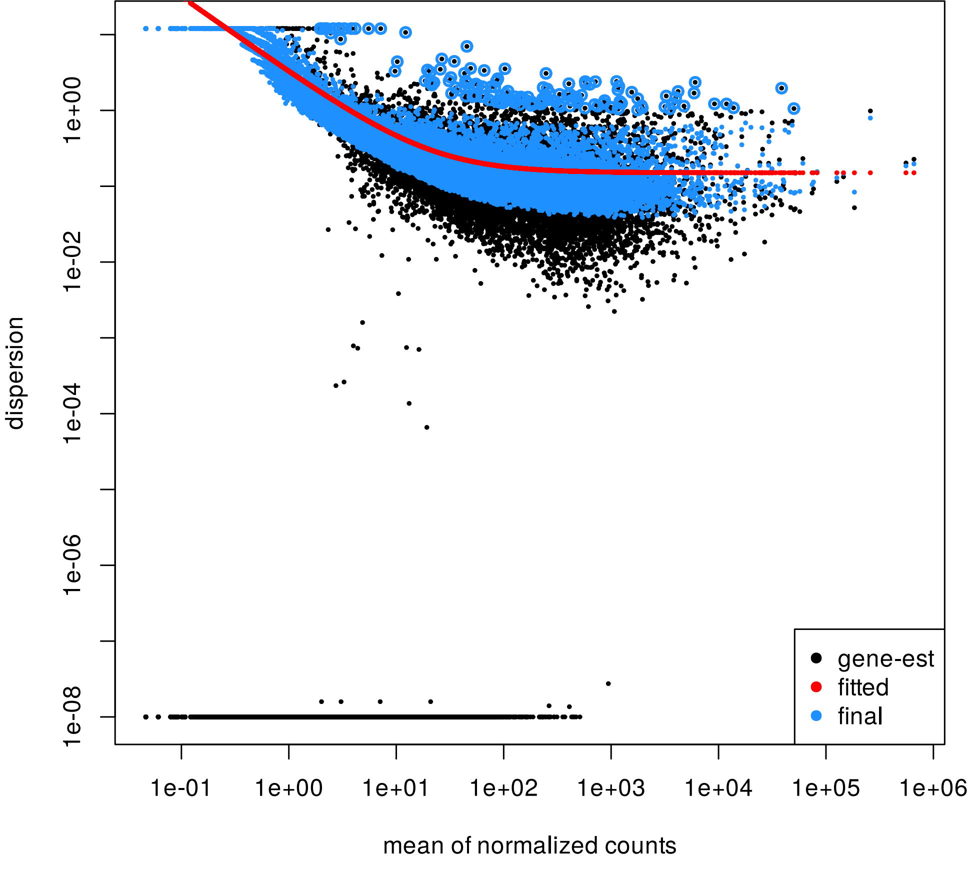

Fig. S1. Dispersion estimates versus mean normalized counts for all genes, illustrating model fit and variance structure in the DESeq2 analysis

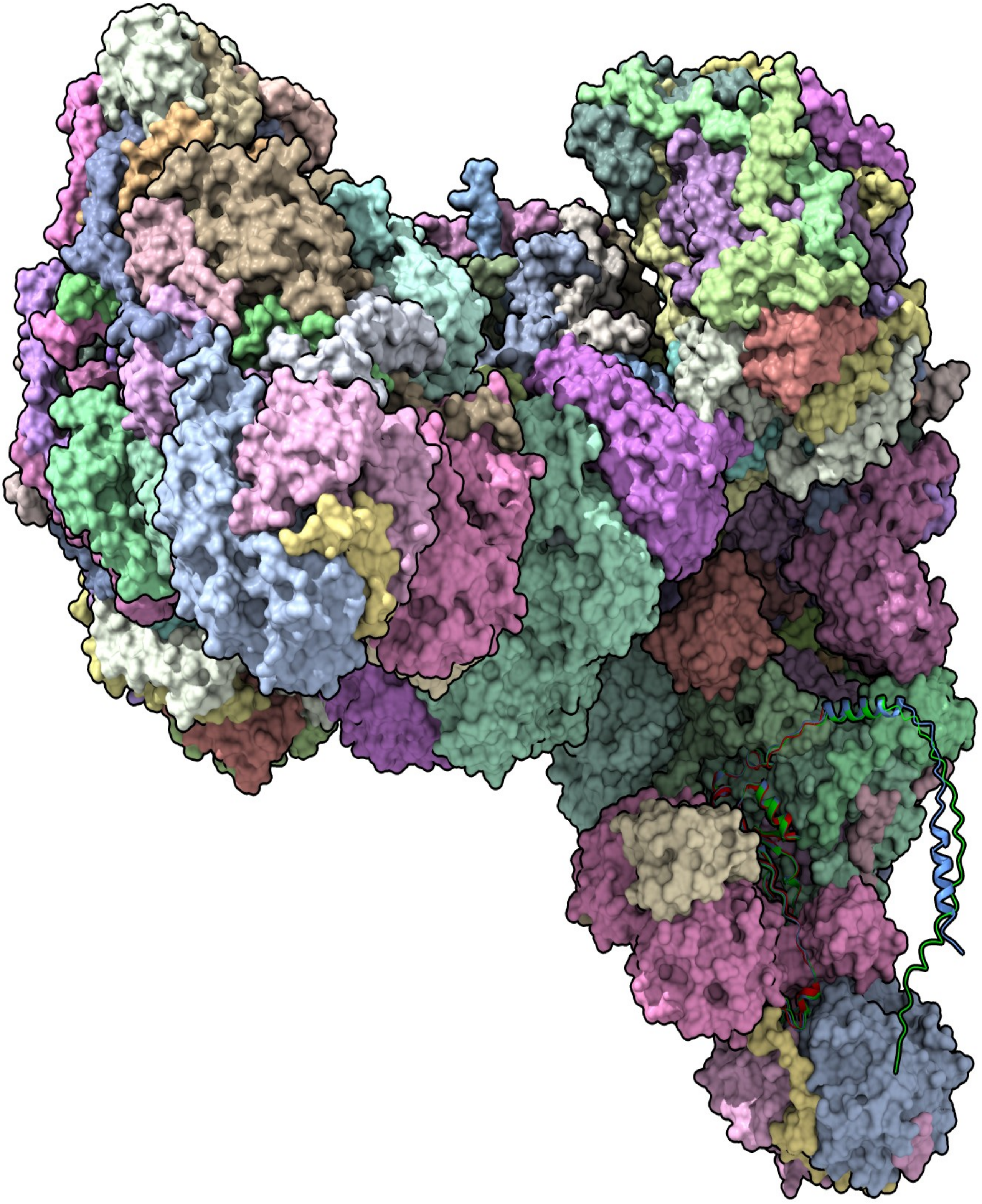

Fig. S2. A molecular model of the mouse (*Mus musculus*) I2+III2 supercomplex derived from cryo-EM 8UCA, not available for human). The predicted secondary structures of the mouse, human, and olm NDUFS4 subunit are shown in red, green, and blue, respectively.

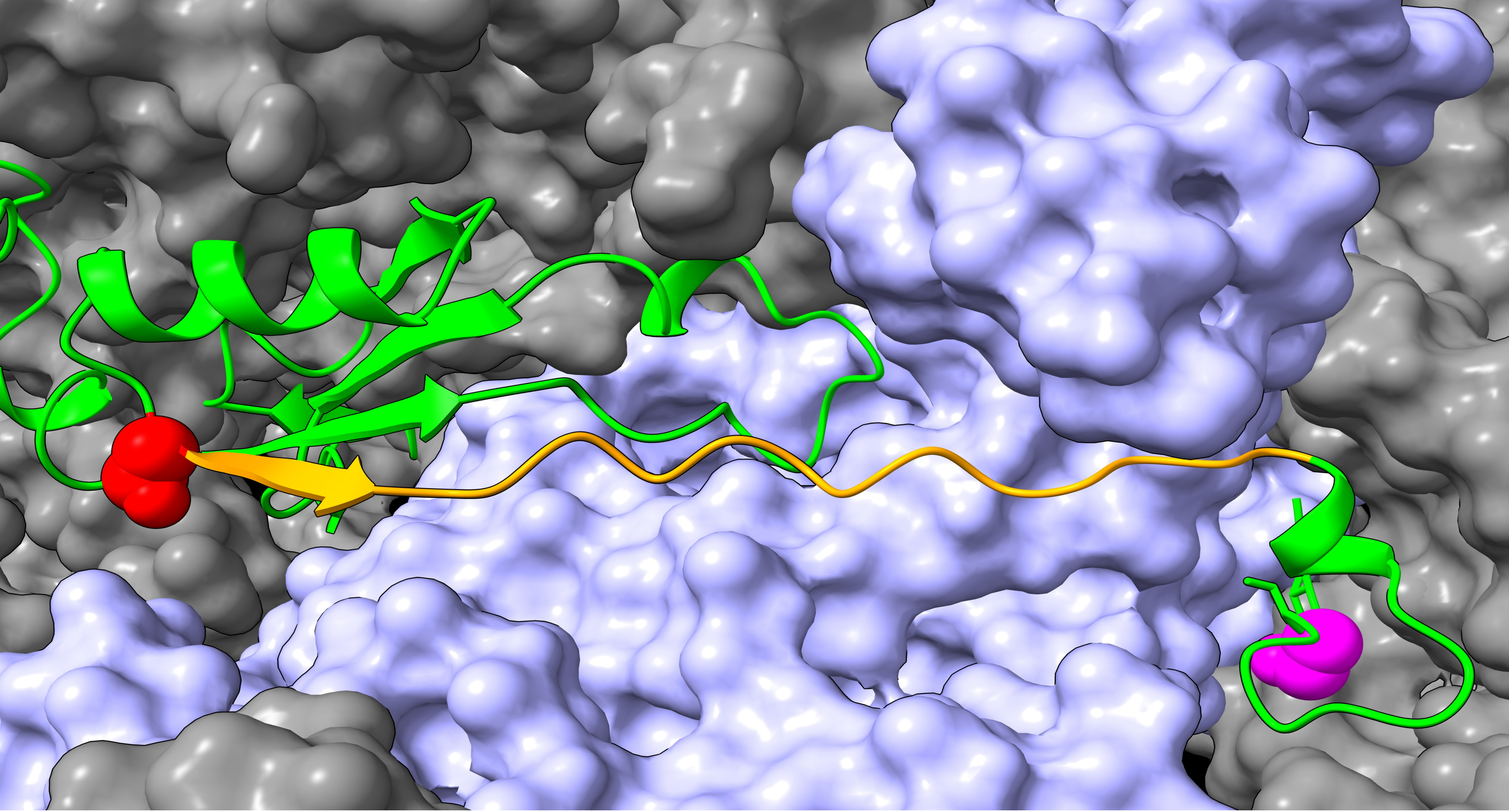

Fig. S3. Model of human NDUFS4 binding to the I2+III2 supercomplex. The conserved site S144 is depicted by red spheres and located at the beginning of a three residue ß-strand. Other residues of this ß-strand and of the extended stretch connecting with the phosphorylation site S173 (magenta) are colored in orange. .
